# Supplementary material for: Dynalign II: common secondary structure prediction for RNA homologs with domain insertions
Source: Nucleic Acids Res. 2014 Nov 21;42(22):13939–48. doi: 10.1093/nar/gku1172 (PMC4267632; doi:10.1093/nar/gku1172)
Supplement: SUPPLEMENTARY DATA [file supp_gku1172_nar-02021-z-2014-File012.zip › manual/javadoc/RNAstructure_java_interface/source/startup/package-summary.html]

RNAstructure\_java\_interface.source.startup


JavaScript is disabled on your browser.


- Overview
- Package
- Class
- Tree
- Index
- Help

- Prev Package
- Next Package

- Frames
- No Frames

- All Classes

# Package RNAstructure\_java\_interface.source.startup

A package that holds all Java RNAstructure interface classes that are directly called at the Java interface startup.

See: Description

- Class Summary

  | Class | Description |
  | --- | --- |
  |  |  |
  | --- | --- |
  | ApplicationRootFrame | A class that creates the application frame of the RNAstructure GUI. |
  | RNAstructure | A class that starts the RNAstructure interface. |
  | RNAstructureBackendCalculator |  |
  | RNAstructureBackendCalculatorProxyJNI |  |


## Package RNAstructure\_java\_interface.source.startup Description

A package that holds all Java RNAstructure interface classes that are directly called at the Java interface startup.
  
  
The RNAstructureBackendCalculator classes are generated by SWIG, and connect the C++ back end to the Java front end.
Neither of those classes are commented.
This is because the SWIG documentation framework is deprecated, and no changes have been made to the generated files, for the sake of clarity and simplicity.
To see the documentation of the C++ classes that back these Java classes (they have the same name as the Java classes), please see the Doxygen documentation for them.

- Overview
- Package
- Class
- Tree
- Index
- Help

- Prev Package
- Next Package

- Frames
- No Frames

- All Classes
